# Impact of multidomain lifestyle intervention on dynamics of cognitive frailty: post hoc analysis of the FINGER trial

**DOI:** 10.1093/gerona/glaf275

**Published:** 2025-12-15

**Authors:** Johanna Pöyhönen, Hanna-Maria Roitto, Jenni Lehtisalo, Esko Levälahti, Timo Strandberg, Miia Kivipelto, Jenni Kulmala, Riitta Antikainen, Hilkka Soininen, Jaakko Tuomilehto, Tiina Laatikainen, Tiia Ngandu

**Affiliations:** Department of Public Health, Finnish Institute for Health and Welfare (THL), Helsinki, Finland; Department of Medicine, Clinicum, University of Helsinki, Helsinki, Finland; Department of Medicine, Clinicum, University of Helsinki, Helsinki, Finland; Department of Geriatrics, Helsinki University Hospital, Helsinki, Finland; Department of Public Health, Finnish Institute for Health and Welfare (THL), Helsinki, Finland; Institute of Clinical Medicine/Neurology, University of Eastern Finland, Kuopio, Finland; Department of Public Health, Finnish Institute for Health and Welfare (THL), Helsinki, Finland; Department of Medicine, Clinicum, University of Helsinki, Helsinki, Finland; Center for Life Course Health Research/Geriatrics, University of Oulu, Oulu, Finland; Division of Clinical Geriatrics, Center for Alzheimer Research, Care Sciences and Society (NVS), Karolinska Institutet, Stockholm, Sweden; Ageing Epidemiology Research Unit (AGE), School of Public Health, Imperial College London, London, United Kingdom; Department of Public Health, Finnish Institute for Health and Welfare (THL), Helsinki, Finland; Faculty of Social Sciences (Health Sciences) and Gerontology Research Center (GEREC), Tampere University, Tampere, Finland; Center for Life Course Health Research/Geriatrics, University of Oulu, Oulu, Finland; Medical Research Center Oulu, Oulu University Hospital, Oulu, Finland; Institute of Clinical Medicine/Neurology, University of Eastern Finland, Kuopio, Finland; Neurocenter, Department of Neurology, Kuopio University Hospital, Kuopio, Finland; National School of Public Health, Madrid, Spain; Department of Public Health, University of Helsinki, Helsinki, Finland; Institute of Public Health and Clinical Nutrition, University of Eastern Finland, Kuopio, Finland; Wellbeing Services County of North Karelia (Siun sote), Joensuu, Finland; Department of Public Health, Finnish Institute for Health and Welfare (THL), Helsinki, Finland; Institute of Public Health and Clinical Nutrition, University of Eastern Finland, Kuopio, Finland

**Keywords:** Clinical trials, Mild cognitive impairment, Frailty, Cognition

## Abstract

**Background:**

Cognitive frailty (CF), a condition with physical frailty and mild cognitive impairment (MCI) without dementia, is potentially reversible and linked to adverse outcomes. We aimed to investigate the impact of a multidomain lifestyle intervention on temporal dynamics of CF in older adults at risk of dementia.

**Methods:**

In the Finnish Geriatric Intervention Study to Prevent Cognitive Impairment and Disability (FINGER), 1259 participants, aged 60-77, were randomized to a 2-year multidomain lifestyle intervention or standard health advice. Frailty was defined by the modified Fried phenotype, and MCI by the lowest quintile in the neuropsychological test battery *z* score. Having pre-frailty/frailty and MCI was classified as CF. Transition probabilities and predictiveness of CF by 4 different baseline groups (healthy, MCI, pre-frail/frail, CF) were examined using multinomial logistic regression.

**Results:**

At baseline, 219 participants (18%) had CF. The risk for developing CF at 2 years was higher in the control group (risk ratio [RR], 1.88, *p* = .003). The intervention effect was not modified by baseline CF (*p* = .493). Reversal from CF to no-CF group was more likely in the intervention group, and progression to or persisting with CF was more likely in the control group. Compared with healthy participants (*n* = 401) at baseline, the MCI group (*n* = 244) had an RR of 5.10, pre-frail/frail (*n* = 336) of 3.06, and CF of 30.61 for having CF at 2 years, with no difference between MCI and pre-frail/frail groups (*p* = .116).

**Conclusions:**

The 2-year multidomain lifestyle intervention was effective in preventing and reversing CF. Participants with MCI or pre-frailty/frailty were both at increased risk for CF compared with healthy participants.

## Introduction

Maintaining independence and functional ability with age relies on preservation of both physical and cognitive capacities. Cognitive frailty (CF), defined as the concurrent presence of physical frailty and mild cognitive impairment (MCI),^1^ is regarded as a phase between normal aging and the development of more severe impairments such as physical disability and/or dementia.^2^^,^^3^

A recent meta-analysis estimated the prevalence of CF at 12.2% among community-dwelling older adults, although reported frequencies vary widely—from 1% to 52%—depending on the definition of CF used and the characteristics of the study population.^4^ Prevalence of frailty is estimated to be around 10%^5^ and MCI around 15%.^6^ The potential mechanisms underlying physical frailty include chronic systemic inflammation, oxidative stress, dysregulation of the hypothalamic–pituitary axis, endocrine disturbances, and mitochondrial dysfunction, all of which contribute to the body’s ability to maintain physiological homeostasis.^7^^,^^8^ Notably, these mechanisms are also associated with cognitive decline and dementia.^7–9^ Physical frailty and cognitive impairment frequently coexist and share not only pathophysiological pathways but also similar adverse outcomes such as increased risk of falls, disability, hospitalization, and mortality.^10^

Cognitive frailty is considered reversible or potentially reversible, particularly when it involves pre-frailty and subjective memory complaints or MCI.^11^^,^^12^ The reversibility underscores the value of early interventions that simultaneously target both physical and cognitive components. Evidence suggests a bidirectional relationship between physical frailty and cognitive impairment^13^: Frailty has been shown to predict cognitive decline,^14–17^ while cognitive impairment may also predict frailty.^18^ A hierarchical study examining the development of physical frailty and cognitive impairment found that co-occurrence of these conditions was associated with the highest risk for incident dementia during follow-up compared with either condition alone. Of note, individuals who developed frailty before cognitive impairment had the lowest risk.^19^ Another hierarchical study reported similar findings, showing that co-occurrence of the 2 conditions was associated with a higher risk for adverse cardiovascular outcomes and mortality than either condition alone.^20^

Only a few studies have examined the effects of a lifestyle intervention on CF,^21^^,^^22^ and those using a multidomain approach are particularly scarce.^22–24^ The *Finnish Geriatric Intervention Study to Prevent Cognitive Impairment and Disability (FINGER)* has demonstrated that a multidomain lifestyle intervention can improve cognitive performance^25^ and prevent or reverse pre-frailty/frailty,^26^^,^^27^ providing a valuable opportunity to explore the concept of CF. The aim of this study was to investigate the impact of a multidomain lifestyle intervention on the temporal dynamics of CF, that is its potential prevention or reversal, from baseline to the 2-year follow-up. Additionally, we aimed to examine the temporal development of physical pre-frailty/frailty and MCI, and their predictive value for the emergence of CF.

## Methods

### Study design and participants

We used data from the FINGER trial, which is a multidomain randomized controlled lifestyle intervention study.^25^ The active 2-year intervention period was conducted during 2009-2014. Altogether 1259 Finnish adults, aged 60-77 years, were recruited from the general population.^28^ The selection of participants was based on the Cardiovascular Risk Factors, Ageing and Dementia risk score of ≥6,^29^ indicating an elevated risk for dementia. Additionally, participants’ cognitive performance, assessed using the Consortium to Establish a Registry for Alzheimer’s Disease, was at or slightly below the mean level for their age in the Finnish population. The recruitment took place at 6 study centers across Finland. In this study, we included participants with complete baseline data for frailty and the neuropsychological test battery (NTB) total *z* score.

### Study protocol

The detailed study protocol has been described elsewhere.^28^ Participants were randomized in a 1:1 ratio to either a multidomain lifestyle intervention group or a control group. The intervention comprised 4 main components: nutrition advice (3 individual and 7-9 group sessions); exercise (individually tailored programs for muscle strength training, aerobic exercise, and exercises to improve postural balance); cognitive training and social activity (included individual computer-based sessions and ten group sessions); and monitoring of metabolic and vascular risks (based on national evidence-based guidelines). The control group received standard health advice in line with national recommendations.

Participants attended annual study visits with a study nurse during the 2-year intervention period. Neuropsychological test battery assessments were conducted annually by a psychologist during the active intervention period (at baseline, year 1, and year 2, ie, post intervention) to evaluate the primary outcome of the trial. A medical examination, including a review of detailed medical history, was performed by a physician at screening and at 2 years. Participants completed a health questionnaire annually. Physical performance measures, including the Short Physical Performance Battery and grip strength, were evaluated at baseline and at 2 years. Relevant nutrient intakes were calculated from 3-day food records at baseline and at 2 years.

The FINGER study was approved by the Coordinating Ethics Committee of the Hospital District of Helsinki and Uusimaa (94/13/03/00/2009). The study was conducted according to the principles of the Declaration of Helsinki, and written informed consent was obtained from all participants at the start of the study and at follow-up visits. This study is registered at ClinicalTrials.gov (no. NCT01041989).

For the present study, we used baseline and longitudinal (2-year) data of frailty status, NTB total, and cognitive subdomain *z* scores to construct the main outcome variable of CF. As covariates, we used randomization group (intervention vs control), Apolipoprotein E (APOE) genotype dichotomized as Ɛ4 carrier versus non-carrier, sex, years of education, baseline age, body mass index (BMI), protein intake (g/kg), and number of chronic diseases (diagnosed or treated in the past 12 months based on a medical history questionnaire filled out by a physician after the participant interview).

### Definition of phenotypic frailty

Phenotypic frailty was assessed via the modified Fried criteria,^30^ with 1 point assigned to each of the 5 components fulfilling the criteria. *Weight loss* was defined as self-reported loss of weight (≥4.5 kg or ≥5%) over the past year. *Weakness* was measured with hand grip strength (the maximum result from 5 measurements of each hand) using a hydraulic hand dynamometer and using the original sex- and BMI-adjusted cut-offs by Fried (men BMI [kg/m^2^] ≤24: ≤29 kg, BMI 24.1-26: ≤30 kg, BMI 26.1-28: ≤30 kg, BMI >28: ≤32 kg; women BMI ≤23: ≤17 kg, BMI 23.1-26: ≤17.3 kg, BMI 26.1-29: ≤18 kg, BMI >29: ≤21 kg).^30^  *Exhaustion* was defined by a question regarding a feeling of weakness or tiredness during the previous month; participants reporting “quite a lot” or “very much” were considered to have exhaustion. *Low physical activity* was evaluated by asking the question: “How often do you in your leisure time exercise for at least 20 minutes so that you are at least mildly out of breath and sweaty?”. Responses of once a week or less, or inability to exercise due to disability or disease, indicated low physical activity. ***Slowness*** was determined from sex- and height-adjusted gait speed (the best result from two 4-m walks at normal walking speed), using cut-offs adapted from Fried’s original 15-feet criteria for 4 m (men ≤173 cm: ≥6.15 s, >173 cm: ≥5.26 s; women ≤159 cm: ≥6.15 s, >159 cm: ≥5.26 s).^30^

Frailty status was classified according to the number of modified Fried criteria met: robust 0 points, pre-frail 1-2 points, and frail 3-5 points. For the analyses, frailty status was dichotomized (robust vs pre-frail/frail) due to the limited number of frail individuals. To address missing data, the following rules were applied: (1) if a participant had at least 1 frailty component scored as positive (1 point), they were categorized as pre-frail/frail, even if data were missing for 1 or more other components (*n* = 48 at baseline, *n* = 59 at 2 years); (2) if the participant scored zero points but had missing data for 1 or more components, frailty status was considered as missing (*n* = 58 at baseline, *n* = 89 at 2 years).

### Cognition assessment

The overall cognitive performance was assessed using the NTB total score, which served as the primary outcome of the FINGER trial.^25^ The NTB total score is a composite *z* score calculated from 14 cognitive tests, with higher scores demonstrating better performance. Standardization was based on the baseline mean and *SD*. In addition to the overall score, domain-specific NTB z scores were calculated for executive function, processing speed, and memory. The content of these tests has been described elsewhere.^25^ Cognitive assessments were performed by a psychologist at baseline and at 2 years. To identify MCI, participants scoring in the lowest quintile (similar approach as used in previous studies^19^^,^^20^) of the NTB total score or any of the cognitive domains at baseline were classified as having MCI. This approach captures both global and domain-specific cognitive decline. The same cut-off values, derived from baseline distributions, were applied at 2 years to assess progression or reversibility of MCI. When the participants had a missing value on the NTB total score or any domain and scored in the upper 80% in other cognition parts, they were classified as missing (*n* = 2 at 2 years). If the participant scored within the lowest quintile on the NTB total score or any domain, they were classified as having MCI even if data were missing for total or other domains (*n* = 1 at baseline, *n* = 7 at 2 years). Because there is no uniform definition of the MCI part of CF, and our definition is based on cognitive tests that can be influenced by education, additional analyses were conducted using education-specific cut-offs for the lowest quintile. The education categories were <8 years, 8-9 years, and ≥10 years to balance group sizes and considering the Finnish school system at the time. This allowed for a more nuanced evaluation of MCI classification relative to participants’ cognitive reserve proxied by education.

### Cognitive frailty

Cognitive frailty was defined as the simultaneous presence of pre-frailty/frailty and MCI. Cognitive frailty status was treated as a dichotomous variable (CF vs no CF) at baseline and a 3-category variable (CF, no CF, or missing data) at 2 years.

### Statistical analyses

Baseline characteristics are presented as means ± *SD*s for continuous variables and as frequencies (%) for categorical variables. Group comparisons were performed using Student’s *t*-test, Mann–Whitney *U*-test, or Fisher’s exact test as appropriate based on data distribution and variable type.

The primary outcome was post-intervention CF status categorized into 3 groups (CF, no CF, and missing data). The participants with missing CF data at the 2-year follow-up on CF were retained as a distinct category to account for possible non-randomness of missing data. A multinomial logistic regression model was used to analyze the intervention effect on CF. The interaction term between randomization groups and baseline CF was added to the model, and marginal effects estimation was used to predict the probabilities of each CF status category at 2 years and to estimate differences in these probabilities between intervention and control groups to provide additional information to the primary analysis. Analyses were adjusted for sex, age, baseline number of chronic diseases (continuous), years of education, baseline protein intake, and study site. To maximize use of available data on CF status, we applied mean imputation to missing values in covariates (baseline protein intake, diseases, and education years; maximum number of imputations *n* = 16). The same methods were applied using an education-stratified definition of CF. For sensitivity analysis, because pre-frail and frail participants were grouped for analyses and the intervention may be more effective at the early stage of frailty, participants who were frail at baseline (*n* = 15) were excluded to evaluate the robustness of the findings.

For cross-sectional comparison, the Sankey flow diagram, and predictiveness analyses, participants were categorized at baseline and at 2 years into a 4-category mutually exclusive variable based on their physical frailty and cognitive status: (1) healthy (robust and no MCI); (2) MCI (robust and MCI); (3) pre-frail/frail (pre-frailty/frailty and no MCI); and (4) CF (pre-frailty/frailty and MCI). Logistic regression was used to analyze the predictive value of each baseline group for CF status at 2 years. In addition, an interaction term between this baseline 4-category variable and the randomization group was included to determine whether any subgroup had greater benefit from the intervention.

*P*-value of <.05 was considered statistically significant, and results are reported with 95% CIs. Statistical analyses were performed using SPSS 29.0 for Windows (SPSS Inc., Chicago, IL, United States) and Stata 18.0 software. RStudio (version 2024.04.1, R Foundation, Vienna, Austria) was used to create the Sankey diagram.

## Results

**Figure 1.**
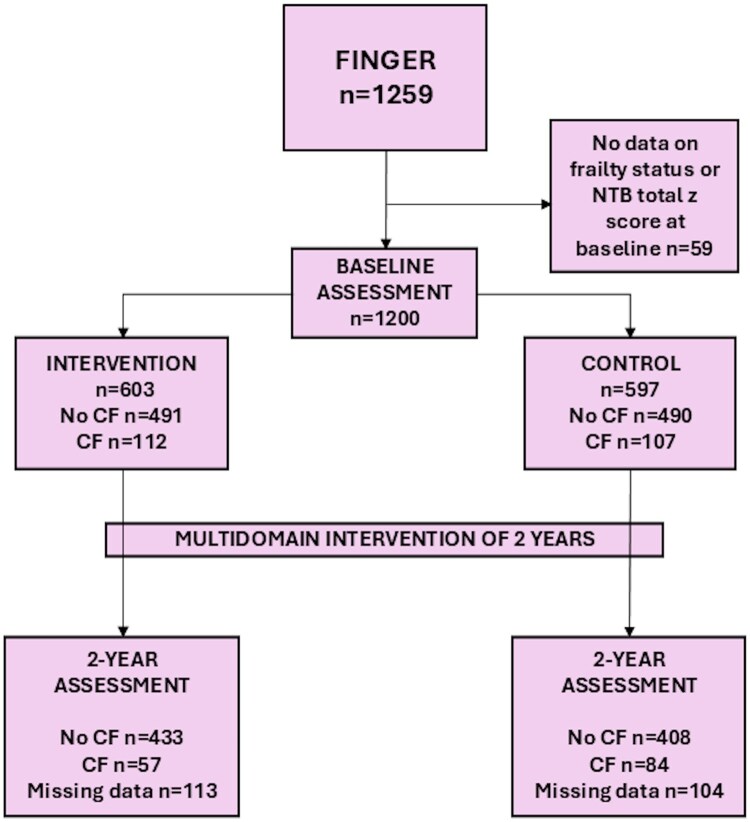
Flow chart. A total of 1200 of 1259 participants in the FINGER trial with baseline data on frailty and cognition were selected for the present study. The flow chart is showing the observed numbers of CF, non-CF groups at baseline and at the 2-year follow-up, and also number of missing data on CF at the 2-year follow-up. CF, cognitive frailty; NTB, neuropsychological test battery.

Of the 1259 participants enrolled in the FINGER study, 1200 (95%) had available data on baseline frailty status, and NTB total *z* score and were included in the analyses (Figure 1). At baseline, the mean age of participants was 69 years, and 550 (46%) were female. Of all participants, 219 (18%) had CF at baseline. Compared with participants without CF, those with CF were slightly older and had fewer years of education, higher BMI, more chronic diseases, and lower protein intake (Table 1). Among participants without CF, those in the control group were younger than those in the intervention group. No other significant differences were observed between intervention and control groups within baseline CF subgroups.

**Table 1.** Baseline characteristics of participants: comparison between groups with and without cognitive frailty (CF).

Characteristics		**All** (*n* = 1200)	**No CF** (*n* = 981)	**CF** (*n* = 219)	*P*-value
**Intervention group**	1200	603 (50.2)	491 (50.1)	112 (51.1)	.823
**Sociodemographics**					
** Age (years)**	1200	69.3 ± 4.7	69.0 ± 4.7	70.8 ± 4.5	**<.001**
** Female sex**	1200	550 (45.8)	447 (45.6)	103 (47.0)	.708
** Education (years)**	1199	10.0 ± 3.4	10.2 ± 3.6	8.8 ± 2.6	**<.001**
**Health factors**					
** MCI**	1200	463 (38.6)	244 (24.9)	219 (100.0)	**<.001**
** Pre-frail or frail**	1200	555 (46.3)	336 (34.3)	219 (100.0)	**<.001**
** Body mass index (kg/m²)**	1196	28.2 ± 4.7	28.0 ± 4.6	29.3 ± 5.1	**<.001**
** Diseases (count)^a^**	1194	2.5 ± 1.5	2.4 ± 1.5	2.9 ± 1.6	**<.001**
** None**		111 (9.3)	101 (10.4)	10 (4.6)	
** 1**		226 (18.9)	194 (19.9)	32 (14.6)	
** 2**		312 (26.1)	259 (26.6)	53 (24.2)	
** ≥3**		545 (45.6)	421 (43.2)	124 (56.6)	
** APOE Ɛ4 carrier (yes)^b^**	1118	363 (32.5)	302 (33.0)	61 (30.2)	.507
** Protein intake (g/kg)**	1190	0.99 ± 0.33	1.00 ± 0.33	0.93 ± 0.32	**.005**

All participants with baseline frailty status and NTB total *z* score data were included in the study. Data are numbers (percentages) of participants or means ± *SD*. Analyses conducted with Fisher’s exact test or non-parametric test. Statistically significant p-values are bolded.

Abbreviations: APOE, Apolipoprotein E; CF, cognitive frailty; MCI, mild cognitive impairment; NTB, neuropsychological test battery.

a Mean number of 18 diagnoses (high blood pressure, heart failure, angina pectoris, cancer, asthma, pulmonary emphysema or chronic bronchitis, angioplasty, coronary bypass, gallstones or gall bladder inflammation, rheumatoid arthritis, other articular disease, back condition, chronic urethritis or nephritis, cerebrovascular disease, diabetes, depression and other psychological illnesses, and other possible chronic disease); self-reported at baseline if diagnosed or treated by a physician during last 12 months.

b Carrier of at least 1 APOE Ɛ4 allele versus non-carriers.

The overall risk for CF at 2 years was significantly higher in the control group than in the intervention group (risk ratio [RR]: 1.88; 95% CI: 1.24-2.84; *p* = .003) (Table S1). There was no statistically significant interaction between baseline CF status and randomization group, indicating that the intervention effect was not modified by initial CF status (*p* = .493 for interaction) (Table S2). Participants in the control group were more likely to transit from no CF at baseline to CF at 2 years with a between-group difference of 2.7 percentage points (95% CI: 0.1-5.4; *p* = .042) (Table 2). Also, the probability of reversing from CF to no CF group was higher in the intervention group (between-group difference −16.6 percentage points; 95% CI: −30.4 to −2.8; *p* = .018), while remaining in the CF group was more likely in the control group (between-group difference 14.1 percentage points; 95% CI: 2.0-26.1; *p* = .022). Sensitivity analyses excluding participants with frailty at baseline (*n* = 15) yielded similar results, confirming the robustness of the findings (Table S3).

**Table 2.** Estimated probabilities of transitions from baseline cognitive frailty (CF) status (no CF or CF) to 2 years (CF status: no CF, CF, or no data).

Baseline CF status, *n* = 1200	Two-year CF status	All participants (%)	Intervention (%)	Control (%)	Difference between groups (95% CI)	*P*-value
**No CF**	No CF	78.6	79.3	77.6	−1.6 (−6.8 to 3.6)	.540
***n* = 981**	CF	5.0	3.8	6.5	2.7 (0.1-5.4)	**.042**
	No data	16.4	16.9	15.8	−1.1 (−5.8 to 3.6)	.640
**CF**	No CF	42.7	51.0	34.5	−16.6 (−30.4 to −2.8)	**.018**
***n* = 219**	CF	29.9	23.2	37.3	14.1 (2.0-26.1)	**.022**
	No data	27.4	25.7	28.3	2.5 (−9.7 to 14.8)	.687

Estimated mean proportions and mean difference derived from the multinomial logistic regression model including an interaction term between baseline cognitive frailty status and randomization group. The model was adjusted for baseline age, sex, education (years), number of chronic diseases, protein intake (g/kg), and study site. Statistically significant p-values are bolded.

Abbreviation: CF, cognitive frailty.

**Figure 2.**
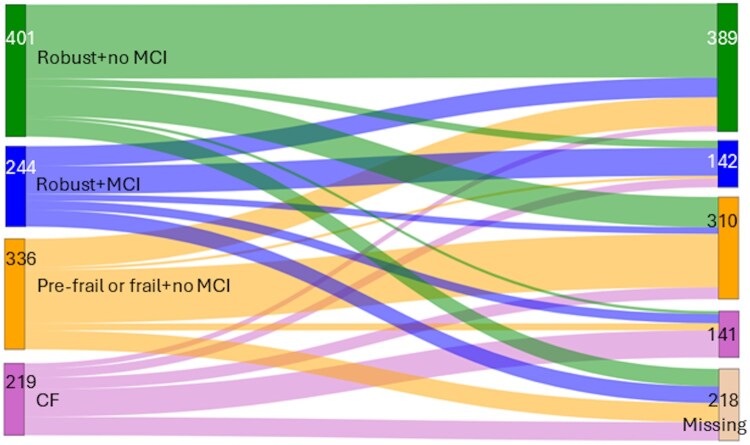
A Sankey diagram showing the transition of participants between cognitive and physical frailty states from baseline to the 2-year follow-up. The figure visualizes the flow of all participants (*n* = 1200) with available baseline frailty status and NTB total *z*-score data through 4 mutually exclusive baseline categories: Healthy (no MCI or pre-frailty/frailty), MCI only, pre-frail/frail only, and CF. Transitions to the corresponding categories at 2 years are shown, including participants lost to follow-up or with missing data. The width of each flow is proportional to the number of participants transitioning between categories. This diagram illustrates the dynamic nature of CF and highlights both progression and reversibility. CF, cognitive frailty; MCI, mild cognitive impairment; NTB, neuropsychological test battery.

The observed transitions among the 4 mutually exclusive participant groups (healthy, MCI, pre-frail/frail, and CF) during the study period are presented in Figure 2. A total of 218 participants (18.2%) were lost to follow-up during the study, with attrition most common among those classified as CF or MCI at baseline. Participants who had CF at baseline were more likely to transit to missing data than healthy participants (25.6% vs 13.2%, *p* < .001). Among the 982 participants (81.8%) who completed the 2-year follow-up, 46.3% maintained the same CF status throughout the study. The most common transitions were from pre-frail/frail to healthy (25.9%) and from MCI to healthy (24.6%). Direct transitions from healthy to CF (2.2%) or from CF to healthy (7.8%) were rare. Throughout the study period, 266 participants (22.2%) developed MCI, and 428 (35.7%) developed pre-frailty/frailty as the first condition. In multinomial logistic regression analyses and using the healthy group as a reference, the RRs for predicting CF at 2 years were higher for the MCI group (RR: 5.10; 95% CI: 2.29-11.37; *p* < .001), the pre-frail/frail group (RR: 3.06; 95% CI: 1.36-6.88; *p* = .007), and the CF group (RR: 30.61; 95% CI: 14.35-65.28; *p* < .001) (Table 3). The control group had a higher risk of CF at the 2-year follow-up (RR: 1.95; 95% CI: 1.29-2.96; *p* = .002). No statistically significant interaction emerged between this 4-category variable and the randomization group (*p* = .462), indicating that the intervention effect was consistent across all 4 baseline categories. Having MCI at baseline did not predict 2-year CF more strongly than having pre-frailty/frailty (RR for MCI vs pre-frailty/frailty 1.67; 95% CI: 0.88-3.15; *p* = .116).

**Table 3.** Risk ratios for different baseline frailty status and mild cognitive impairment combinations predicting cognitive frailty over 2 years.

Baseline	RR (95% CI)	*P*-value
**Healthy (reference)**	1	
**MCI**	5.10 (2.29-11.37)	**<.001**
**Pre-frail or frail**	3.06 (1.36-6.88)	**.007**
**CF**	30.6 (14.4-65.3)	**<.001**
**Group (intervention as reference)**	1.95 (1.30-2.96)	**.002**

Multinomial logistic regression model was used to estimate risk ratios for predicting CF at 2 years (no CF as a reference category, and accounting for participants with missing follow-up data). The model assessed 4 mutually exclusive baseline groups based on combinations of frailty status and MCI, with healthy group used as the reference. The model was adjusted for baseline age, sex, randomization group, education (years), number of chronic diseases, protein intake (g/kg), and study site. Statistically significant p-values are bolded.

Abbreviations: CF, cognitive frailty; MCI, mild cognitive impairment; RR, risk ratio.

The additional analyses using education-stratified cognitive cut-offs yielded similar results among the participants with CF at baseline. The overall intervention effect on CF approached statistical significance (*p* = .059). When including the interaction term between randomization group and baseline CF status, a significant beneficial effect was observed, particularly among participants who had CF at baseline. Participants in the control group were more likely to maintain CF (between-group difference 13.4 percentage points; 95% CI: 0.8-26.0; *p* = .037), while those in the intervention group were more likely to reverse from CF to no CF (between-group difference −15.4; 95% CI: −28.9 to −1.9; *p* = .025) (Table S4).

## Discussion

According to our study, the 2-year multidomain lifestyle intervention was effective in both preventing and reversing CF among older adults at risk of dementia. We also found that those with either MCI or pre-frailty/frailty at baseline had an increased risk for developing CF compared with healthy participants over the study period. Although the risk estimate was numerically higher for the MCI group, the difference in CF risk between MCI and pre-frail/frail groups was not statistically significant. Importantly, participants with MCI, pre-frailty/frailty, or CF at baseline appeared to benefit equally from the intervention.

Many previous studies have focused on interventions targeting physical frailty, showing that it can be reduced or prevented through exercise and dietary advice.^31^^,^^32^ Additionally, evidence exists on the prevention of cognitive decline with multidomain lifestyle interventions,^25^ and MCI has been shown to be dynamic, with also reversal possible, along with remaining stable or progressing to more severe impairment.^33^ As frailty and cognitive impairment have possible joint pathophysiological pathways,^8^ beneficial lifestyle intervention effects on CF seem inherent. However, only a few studies have assessed the effect of a lifestyle intervention on CF,^21^^,^^22^ and particularly studies using a multidomain approach are scarce and have shown conflicting results.^22–24^ The AGELESS trial investigated the effect of a 2-year multidomain lifestyle intervention (similar to ours) on participants with CF (Fried phenotype ≥1 and Clinical Dementia Rating [CDR] 0.5 and Mini-Mental State Examination score 19-25).^22^ Unlike our study, they did not find significant differences between groups in reversing CF. However, the trial had a high dropout rate (50%), partly due to the COVID-19 pandemic. Despite this, improvements were seen in several cognitive and physical performance outcomes at 12 months. Notably, intervention visits were less frequent during the second intervention year, and many benefits diminished, highlighting the need for continuous support. Another study using an 8-week virtual reality motor-cognitive training on participants with CF (Fried phenotype ≥1 and MCI defined by Montreal Cognitive Assessment score ≤25 and CDR 0.5) showed a beneficial effect on cognition but not on frailty.^23^ A third study reported that the combination of mindfulness and Tai Chi for 6 months was more effective in reversing CF (Fried phenotype pre-frail or frail and MCI defined by CDR 0.5) than either intervention alone.^24^ Importantly, all 3 aforementioned studies included only participants with CF and thus could not assess progression to CF. Furthermore, differences in CF definitions and intervention durations make direct comparisons challenging. Even though the latter 2 studies can be considered multidomain, our study covered a broader range of healthy lifestyle components. Reviews have shown that a multidomain approach may be more effective concerning the prevention of frailty and cognitive decline,^34^^,^^35^ but targets should be carefully planned to balance effectiveness and equity.^36^

Compared with our study, many previous interventions have had shorter durations or have comprised single-domain approaches. According to a review with meta-analyses, multicomponent exercise programs can improve cognitive function and frailty status in older adults with CF, with moderate certainty of evidence.^37^ Based on this review, for hospitalized older adults with CF, nutritional support was found to be the most effective intervention, while aerobic exercise and dual-task training were also effective. Another review with meta-analyses similarly demonstrated the effectiveness of various interventions for individuals with CF.^21^ However, both of these reviews included a small number of studies, and most studies have evaluated cognition and physical frailty separately, making direct comparison with our results difficult. A 24-month intervention study reported that a physical activity intervention reduced the risk for increasing severity of CF (defined by the Study of Osteoporotic Fractures frailty index and modified Mini-Mental State Examination scale < 88).^38^ Unlike our study, that study treated participants with pre-frailty or frailty without MCI and those with MCI without pre-frailty or frailty as milder stages of CF. The participants in that study were older than in our study, and the intervention focused on a single domain. Another study with a 4-month high-speed resistance exercise training in participants with CF (Fried phenotype pre-frail or frail and CDR 0.5) showed improvements in cognitive function and physical performance, but no significant effect was found on frailty score.^39^

The predictiveness of CF over 2 years was not statistically different between baseline pre-frail/frail and MCI groups, although the risk estimate was higher for MCI. Two earlier hierarchical studies have investigated the occurrence patterns and etiological pathways of phenotypic frailty and cognitive impairment in relation to incident dementia^19^ and cardiovascular risk^20^ over 5 years. Similar to our study, both studies used the lowest quintile in cognitive performance assessment (in at least 1 of 2 cognitive domains: executive functioning and memory). In addition, they included self- or proxy-reported physician diagnosis of dementia and/or score ≥2 on the AD8, an 8-item tool to detect dementia, as indicators of cognitive impairment. Frailty was defined by Fried phenotype criteria with ≥3 components. In both studies, cognitive impairment was more commonly developed first. In contrast, in our study, pre-frailty/frailty emerged first in 36% of participants, while MCI developed first in 22%. This difference may be partly explained by most of the physically frail participants in our cohort being classified as pre-frail. One of the prior studies found that participants with co-occurring frailty and cognitive impairment had the highest risk for developing dementia, while those who developed frailty first had the lowest risk.^19^ The authors hypothesized that when cognitive impairment co-occurs with or precedes frailty, dementia-related pathologies may drive the development of frailty. Conversely, frailty preceding cognitive impairment may reflect a distinct etiological pathway, potentially linked to vascular or inflammatory mechanisms. It is also known that cardiovascular risk factors at midlife predict frailty.^40^ However, as noted in a recent review, distinguishing whether cognitive impairment is caused by neurodegenerative disease or vascular risk factors remains challenging in both clinical and research contexts.^41^ Indeed, most older adults with MCI are found to have mixed pathologies. Supporting the concept of etiological heterogeneity, a longitudinal observational study showed that reversible CF is a short- and long-term predictor of overall dementia, particularly vascular dementia.^42^ Based on our findings, individuals with either MCI or pre-frailty/frailty benefit equally from a multidomain lifestyle intervention in the prevention or reversal of CF. This suggests that targeting both cognitive and physical domains early may delay progression toward more severe functional impairments and dementia.

Our study has some limitations. At the 2-year follow-up, the number of participants with CF was relatively small (*n* = 141), resulting in wide confidence intervals and limited statistical power. This particularly affected analyses involving the interaction between 4-category baseline CF status variable and randomization group. The 2-year study period can be short for studying CF and having only 2 assessments (pre/post) limits analytic possibilities. Grouping pre-frail and frail was warranted due to limited number of frail participants. Also, missing data at 2 years was 18.2%, but this was taken into consideration in the analyses as 1 group in the outcome variable. Different frailty measures (such as frailty index) may capture different aspects of frailty. We used phenotypic frailty, which was defined in alignment with the original Fried phenotype criteria,^30^ but small modifications were necessary. For instance, weight loss was assessed via self-report without information on whether it was involuntary, which was part of the original Fried definition. Physical activity assessment was assessed using a different method; the original Fried phenotype used estimated kilocalories expended, while we relied on frequency-based self-reporting. Using a modified definition may have affected the predictive value of pre-frailty/frailty.^43^ As CF has no agreed-upon gold-standard measures, we assessed MCI with NTB, which is known to be reliable and sensitive measurement on cognition in clinical trials^44^ and was the outcome measure in FINGER trial. Additionally, it is important to note that the original FINGER study was designed with a different primary outcome, cognitive performance, although our CF definition incorporated this outcome. Strengths of this study include a randomized controlled design, the relatively long intervention period, and a large, well-characterized study population. Participants were selected based on an elevated risk for dementia, which is relevant given that frailty and dementia share several risk factors.^8^ Therefore, the FINGER cohort represents an appropriate and valuable population for investigating the prevention and dynamics of CF. The similarity of the findings in education-stratified sensitivity analyses strengthens our main results and help in reducing the possibility of an education-biased MCI definition.

There is no consensus on the clinical utility of the CF concept, and it remains underrecognized among clinicians.^45^ However, accumulating evidence suggests that CF is a meaningful predictor of adverse outcomes, including disability, reduced quality of life, and increased mortality.^41^ Moreover, individuals with CF tend to exhibit worse cognitive performance than those with cognitive impairment alone,^46^ and the co-occurrence of frailty and MCI predicts dementia more than either condition alone.^19^ The definition of reversible CF,^11^ which includes older adults with physical pre-frailty and subjective memory complaints, may offer a practical framework for identifying individuals in early modifiable stages of decline. In this context, our results demonstrating both prevention and reversal of CF with a multidomain lifestyle intervention are promising. Further investigations of the clinical relevance and applicability of CF are warranted. In particular, the concept of reversible CF may provide a valuable target for personalized, multimodal interventions aimed at preventing late-life dementia and functional disability. Extended follow-up studies are needed to evaluate the long-term effectiveness of such interventions and to determine whether CF can serve as a viable clinical tool in preventive geriatrics. Knowledge on possibilities to prevent CF could potentially have a beneficial impact not only on the expected increased burden for healthcare systems but also on the well-being and quality of life of older individuals and their families.

### Conclusions

The 2-year multidomain lifestyle intervention showed effectiveness in reducing the risk of CF. The effect was both reversion and prevention of CF among older adults at risk of dementia. Participants with either pre-frailty/frailty or MCI have an elevated risk for developing CF. Importantly, the intervention appeared to be equally beneficial for the baseline subgroups of MCI, pre-frail/frail, and CF, highlighting its broad applicability in mitigating CF progression and promoting reversibility.

## Supplementary Material

glaf275_Supplementary_Data

## Data Availability

The data presented in this article are not available due to legal and ethical reasons. Complete de-identification is not possible, as this study is part of an ongoing trial. The participants gave informed consent that allows data use only under a confidentiality agreement. The data contain sensitive information, and public data deposition may cause privacy risks. However, researchers who meet the criteria for access to confidential data, as specified by Finnish law and the Finnish Institute for Health and Welfare, may request access following the completion of a material transfer agreement. Data access inquiries should be directed to kirjaamo@thl.fi.
