# Supplementary material for: Impact of multidomain lifestyle intervention on dynamics of cognitive frailty: post hoc analysis of the FINGER trial
Source: J Gerontol A Biol Sci Med Sci. 2025 Dec 15;81(2):glaf275. doi: 10.1093/gerona/glaf275 (PMC12832955; doi:10.1093/gerona/glaf275)
Supplement: glaf275_Supplementary_Data [file glaf275_supplementary_data.docx]

**Impact of Multidomain Lifestyle Intervention on Dynamics of Cognitive Frailty; a Post-hoc Analysis of Finger Trial**

**Table of contents:**

**eTable 1**: Risk ratios from the adjusted multinomial logistic regression model predicting 2-year cognitive frailty (CF) status from baseline.

**eTable 2**: Risk ratios from the adjusted multinomial logistic regression model predicting 2-year cognitive frailty (CF) status from baseline, with interaction between baseline CF status and randomization group

**eTable 3**: Estimated probabilities of transitions from baseline cognitive frailty (CF) status (no CF or CF) to 2 years (CF status: no CF, CF or no data), participants with frailty excluded (n=15).

**eTable 4**: Estimated probabilities of transitions from baseline cognitive frailty (CF) status (no CF or CF) to 2 years (CF status: no CF, CF or no data), using education-specific cut-offs for mild cognitive impairment (MCI).

| **eTable 1. Risk ratios from the adjusted multinomial logistic regression model predicting 2-year cognitive frailty (CF) status from baseline** | | | |
| --- | --- | --- | --- |
|  | **RR (95% CI)** | | ***P*-value** |
|  | No CF | CF |  |
| CF baseline | 1 | 10.9 (7.08–16.84) | **<0.001** |
| Group (intervention as reference) | 1 | 1.88 (1.24–2.84) | **0.003** |
| Sex (female as reference) | 1 | 0.91 (0.61–1.38) | 0.666 |
| Age (years) | 1 | 1.12 (1.07–1.18) | **<0.001** |
| Protein intake baseline (g/kg) | 1 | 0.44 (0.22–0.88) | **0.020** |
| Education (years) | 1 | 0.94 (0.87–1.01) | 0.073 |
| Diseases (category)^a^ | 1 | 1.32 (1.04–1.68) | **0.024** |
| Study site (Helsinki as reference) |  |  | 0.389 |
| Vantaa | 1 | 1.01 (0.45–2.28) | 0.972 |
| Seinäjoki | 1 | 0.89 (0.39–2.05) | 0.790 |
| Oulu | 1 | 1.12 (0.44–2.86) | 0.817 |
| Kuopio | 1 | 1.14 (0.57–2.26) | 0.709 |
| Turku | 1 | 1.80 (0.89–3.63) | 0.103 |
| *Note:* Risk ratios from the adjusted multinomial logistic regression model. Risk estimates account for three possible CF outcomes at 2 years: no CF, CF, and missing data. *Abbreviations: CF=cognitive frailty, RR=risk ratio, CI=confidence interval. ^a^*Categories: 0, 1, 2 and 3 or more diseases. | | | |

| **eTable 2.** **Risk ratios from the adjusted multinomial logistic regression model predicting 2-year cognitive frailty (CF) status from baseline, with interaction between baseline CF status and randomization group** | | | |
| --- | --- | --- | --- |
|  | **RR (95% CI)** | | **P-value** |
|  | No CF | CF |  |
| CF baseline | 1 | 9.51 (5.06–17.87) | **<0.001** |
| Group (intervention as reference) | 1 | 1.76 (1.01–3.07) | **0.047** |
| CF baseline*group | 1 | 1.35 (0.57–3.21) | 0.493 |
| Sex (female as reference) | 1 | 0.91 (0.60–1.38) | 0.657 |
| Age (years) | 1 | 1.12 (1.07–1.17) | **<0.001** |
| Protein intake baseline (g/kg) | 1 | 0.44 (0.22–0.88) | **0.020** |
| Education (years) | 1 | 0.94 (0.87–1.01) | 0.055 |
| Diseases (category)^a^ | 1 | 1.32 (1.04–1.69) | **0.024** |
| Study site (Helsinki as reference) |  |  | 0.395 |
| Vantaa | 1 | 1.02 (0.45–2.29) | 0.969 |
| Seinäjoki | 1 | 0.90 (0.39–2.08) | 0.810 |
| Oulu | 1 | 1.12 (0.44–2.88) | 0.811 |
| Kuopio | 1 | 1.13 (0.57–2.24) | 0.726 |
| Turku | 1 | 1.80 (0.89–3.63) | 0.103 |
| *Note:* Risk ratios from the adjusted multinomial logistic regression model. Risk estimates account for three possible CF outcomes at 2 years: no CF, CF, and missing data. *Abbreviations: CF=cognitive frailty, RR=risk ratio, CI=confidence interval.* ^a^Categories: 0, 1, 2 and 3 or more diseases. | | | |

| **eTable 3. Estimated probabilities of transitions from baseline cognitive frailty (CF) status (no CF or CF) to 2 years (CF status: no CF, CF or no data), participants with frailty excluded (n=15)** | | | | | | |
| --- | --- | --- | --- | --- | --- | --- |
| **Baseline CF status** n=1185 | **2-year CF status** | **All participants (%)** | **Intervention (%)** | **Control (%)** | **Difference between groups (95% CI)** | ***P*-value** |
| **No CF** | No CF | 78.9 | 79.8 | 77.7 | -2.2 (-7.3–3.0) | 0.414 |
| n=973 | CF | 4.7 | 3.4 | 6.5 | 3.0 (0.4–5.6) | **0.022** |
|  | No data | 16.3 | 16.7 | 15.9 | -0.9 (-5.6–3.8) | 0.718 |
| **CF** | No CF | 43.4 | 52.1 | 34.8 | -17.3 (-31.3–(-3.3)) | **0.016** |
| n=212 | CF | 28.2 | 21.6 | 35.6 | 14.1 (2.1–26.1) | **0.021** |
|  | No data | 28.4 | 26.4 | 29.5 | 3.2 (-9.4–15.8) | 0.620 |
| *Note*: Estimated mean proportions and mean difference from the multinomial logistic regression model including interaction between baseline cognitive frailty status and randomization group. The model was adjusted for baseline age, sex, education (years), number of chronic diseases, protein intake (g/kg), and study site. Participants with frailty at baseline (n=15) were excluded from the analyses. *Abbreviations: CF=cognitive frailty, CI=confidence interval.* | | | | | | |

| **eTable 4.** **Estimated probabilities of transitions in baseline cognitive frailty (CF) status (no CF or CF) to 2 years (CF status: no CF, CF or no data), using education-specific cut-offs for mild cognitive impairment (MCI)** | | | | | | |
| --- | --- | --- | --- | --- | --- | --- |
| **Baseline CF^a^ status** n=1199 | **2-year CF status** | **All participants (%)** | **Intervention (%)** | **Control (%)** | **Difference between groups (95% CI)** | ***P*-value** |
| **No CF** | No CF | 78.4 | 78.3 | 78.4 | 0.2 (-5.0–5.3) | 0.952 |
| n=977 | CF | 5.1 | 4.7 | 5.6 | 0.9 (-1.7–3.6) | 0.492 |
|  | No data | 16.5 | 17.1 | 16.0 | -1.1 (-5.8–3.6) | 0.650 |
| **CF** | No CF | 40.8 | 48.7 | 33.2 | -15.4 (-28.9–(-1.9)) | **0.025** |
| n=222 | CF | 33.5 | 27.0 | 40.5 | 13.4 (0.8–26.0) | **0.037** |
|  | No data | 25.7 | 24.3 | 26.3 | 2.0 (-10.0–13.9) | 0.747 |
| *Note:* Estimated mean proportions and mean difference from the multinomial logistic regression model including interaction between baseline cognitive frailty status and randomization group. The model was adjusted for baseline age, sex, education (years), number of chronic diseases, protein intake (g/kg), and study site. *Abbreviations: CF=cognitive frailty, MCI=mild cognitive impairment, CI=confidence interval.* ^a^MCI defined by different cut-offs for 3 education classes (<8, 8–9, ≥10 years) | | | | | | |
